# Novel Compounds Derived from DFPM Induce Root Growth Arrest through the Specific *VICTR* Alleles of Arabidopsis Accessions

**DOI:** 10.3390/life13091797

**Published:** 2023-08-23

**Authors:** Seojung Kim, Miri Cho, Tae-Houn Kim

**Affiliations:** 1 Department of Bio-Health Convergence, Duksung Women’s University, Seoul 01369, Republic of Korea; 2 Department of Biotechnology, Duksung Women’s University, Seoul 01369, Republic of Korea

**Keywords:** DFPM, DFPM derivatives, TNL, ABA signaling, abiotic stress

## Abstract

The small compound [5-(3,4-dichlorophenyl) furan-2-yl]-piperidine-1-ylmethanethione (DFPM) inhibits ABA responses by activating effector-triggered immune signal transduction in Arabidopsis. In addition to the known function of DFPM as an antagonist of ABA signaling, DFPM causes accession-specific root growth arrest in Arabidopsis Columbia-0 via the TIR-NLR protein *VICTR* (VARIATION IN COMPOUND TRIGGERED ROOT growth response) in an EDS1/PAD4/RAR1/SGT1B-dependent manner. Although DFPM could control the specific steps of various cellular responses, the functional residues for the activity of DFPM or the existence of a stronger version of DFPM modification have not been characterized thoroughly. This study analyzed twenty-two DFPM derivatives during root growth arrest, inhibition of ABA signaling, and induction of biotic signal transduction to determine critical residues that confer the specific activity of DFPM. Furthermore, this study identified two more Arabidopsis accessions that generate significant root growth arrest in response to DFPM derivatives dependent on multiple amino acid polymorphisms in the coding region of *VICTR*. The isolation of novel compounds, such as DFPM-5, and specific amino acid polymorphisms critical for the compound-induced responses will help determine the detailed regulatory mechanism for how DFPM regulates abiotic and biotic stress signaling interactions.

## 1. Introduction

Chemical genetics has become a powerful approach using small molecules to dissect complex physiological responses in plants. Small molecules allow the rapid, reversible, and conditional manipulation of biological systems at the subcellular or organismal level [1,2]. The small molecule approach can also overcome the limitations of conventional genetic studies, such as genetic redundancy and lethality problems [3,4]. High-throughput screening of diverse chemical libraries has been validated as a compelling tool to identify novel molecules that regulate many cellular responses, such as plant hormone signaling. The characterization of the specific target proteins and signaling pathways induced by a bioactive small molecule has led to the discovery of novel regulatory components in many phytohormone signaling networks [5,6,7,8]. The plant hormone ABA regulates various physiological processes of plant growth, development, and adaptive responses to diverse environmental stresses [9]. The increased ABAs bind to the RCAR/PYR/PYL receptors, allowing SnRK2s to phosphorylate the downstream transcription factors and the ion channels, leading to cellular responses against stresses [10,11,12]. A small molecule called DFPM ([5-(3,4-dichlorophenyl)furan-2-yl]-piperidine-1-ylmethanethione) that effectively antagonized ABA-responsive gene expression was isolated by screening a 9600-compound chemical library [13]. The inhibitory effects of DFPM were not observed in the defense signaling mutants, including *eds1-2*, *pad4-1*, *sgt1b*, and *rar1-21*, showing that the DFPM inhibition of ABA signal transduction requires early signaling components of effector-triggered immunity (ETI) [13].

Plant disease resistance (*R*) genes usually encode nucleotide-binding and leucine-rich repeat (NLR) receptors that mediate the recognition of diverse effectors from many classes of plant pathogens and activate the ETI responses [14,15,16]. Effector recognition by the NLRs can occur due to either direct physical interactions between the NLRs and the effectors or the monitoring of the modification of host proteins targeted by the effectors [17]. Plant NLRs can be divided mainly into three subfamilies depending on their N-terminal signaling domain: the Toll/interleukin-1 receptor (TIR)-NLR (TNL), the coiled-coil (CC)-NLR (CNL), or the RPW8 (Resistance to Powdery Mildew 8)-like coiled-coil (CC_R_)-NLR (RNL). These receptors influence the requirement for distinct downstream defense signaling components [18,19]. The TIR domains of TNLs have enzyme activities that generate nucleotide metabolites mediating the complex formation of EDS1 (Enhanced Disease Susceptibility 1)-PAD4 (Phytoalexin Deficient 4) and EDS1-SAG101 (Senescence-Associated Gene 101) dimers with ADR1 (Activated Disease Resistance 1) and NRG1 (N Requirement Gene 1), respectively [20,21,22]. A single plant genome usually encodes hundreds of NLR genes to combat diverse and fast-evolving pathogens. These gene numbers and sequence compositions vary substantially among plant species or even within family members in the same genome [23,24]. For example, in the genome of Arabidopsis Col-0 accession, 160 NLR-encoding genes were identified, while up to 251 NLR genes were found in other accessions of Arabidopsis [24,25,26]. More than 500 NLR-encoding genes are annotated in rice cultivars, and over 2000 NLRs are annotated in bread wheat, the largest number reported so far [27,28]. Interestingly, DFPM generates an accession-specific root growth arrest in the Col-0 accession of *Arabidopsis thaliana* via a natural genetic variant in the TNL protein *VICTR* (VARIATION IN COMPOUND TRIGGERED ROOT growth response). Furthermore, the DFPM-mediated root growth inhibition requires the early components of the *R* gene resistance pathway, including *EDS1*, *PAD4*, *RAR1*, and *SGT1B* [29]. DFPM might be involved in controlling several signaling pathways affecting plant stress responses and root growth, considering the multiple phenotypes caused by DFPM and its dependence on certain gene products. Nevertheless, the correlation between its structural motifs and a specific biological activity has not been characterized.

This study analyzed twenty-two derivatives of DFPM to characterize the specific activity of DFPM with modifications of functional residues. The root growth arrest phenotype was initially examined for twenty-two DFPM derivatives, and five candidate compounds with a strong inhibitory effect on the primary root growth were isolated. The five notable compounds were analyzed further by gene expression analyses and root growth assays using defense-signaling mutants and various Arabidopsis accessions. As a result, the derivative DFPM-5 enhanced the root growth arrest and inhibited the ABA-induced gene expression more than the level of the original DFPM. Furthermore, several specific amino acid polymorphisms within the *VICTR* locus are critical for triggering compound-induced root growth inhibition. Novel compounds, such as DFPM-5, might be an efficient tool for further investigating abiotic and biotic stress pathways.

## 2. Materials and Methods

### 2.1. Chemicals

Twenty-two DFPM derivatives were synthesized as described by Lee et al. [30].

### 2.2. Plant Materials and Root Growth Assay 

Seeds of Arabidopsis (Columbia–0 (Col-0) accession) were sterilized with 100% ethanol and sown on growth medium (0.5× Murashige & Skoog (MS), 0.05% MES, 1% sucrose, 0.8% plant agar, pH 5.8). After two days of stratification at 4 °C, the plants were grown vertically for eight days at 22 °C under long-day (16 h light/8 h dark) conditions. Eight-day-old seedlings were transferred to a new solid medium containing 10 µM of each DFPM derivative. The ends of each primary and lateral root of seedlings were marked and monitored to observe root growth arrest after six more days. All root growth experiments were repeated at least three times in independent experiments. The Arabidopsis ecotype set, *eds1-2*, *pad4-1*, *sgt1b*, and *victr1-1* in the Col-0 accession, was described previously [29].

### 2.3. Reporter Gene Expression Analysis

The homozygous T3 transgenic *RAB18:GFP* line was used to examine the inhibitory effects of ABA-induced *RAB18* expression [13]. Transgenic lines were grown vertically for 14 days on agar growth media (0.5× MS), transferred to eight-well culture plates containing 40 µM of each DFPM derivative or DMSO, and incubated for six hours. After pre-treatment with DFPM derivatives, 10 µM ABA was added and incubated for 10 h. The *RAB18* reporter gene expression was monitored using a NIGTHSEA SFA (Electron Microscopy Sciences, Hatfield, PA, USA) light and filter set (Royal blue).

### 2.4. Quantitative Real-Time PCR

Arabidopsis Col-0 seedlings grown for 12 days on growth media were pre-treated with 30 μM DFPM derivatives or DMSO for an hour and then treated with 10 µM ABA for five hours. The total RNAs were extracted using TRIsure™ (Meridian Bioscience Inc., Memphis, TN, USA) and treated with DNase I (Thermo Fisher Scientific, Waltham, MA, USA) before reverse transcription using SensiFAST™ cDNA Synthesis Kit (Meridian Bioscience Inc., Memphis, TN, USA). The mRNA expression of the target gene was measured by RT-qPCR using SensiFAST™ SYBR^®^ Hi-ROX Kit (Meridian Bioscience Inc., Memphis, TN, USA) and normalized relative to the expression of the *Clathrin* gene (At4g24550) (Table S1). Four replicates were run for each sample.

### 2.5. Comparative Sequence Analysis

*VICTR* genomic DNA fragments from 8 *Arabidopsis* accessions (Col-0, Leo1, Nie1.2, Bay-0, Kin-0, Bla1, ICE111, and ICE 112) were amplified using multiple primer pairs (Table S1). The deduced amino acid sequences of the amplified DNA fragments from each Arabidopsis accession were aligned using the Clustal Omega multiple sequence alignment program (https://www.ebi.ac.uk/Tools/msa/clustalo/, accessed on 19 October 2022) [31]. Analyses of DNA similarity were carried out using the BLASTn and BLASTx packages at NCBI (https://blast.ncbi.nlm.nih.gov/Blast.cgi, accessed on 2 November 2022) and TAIR (http://www.arabidopsis.org, accessed on 2 November 2022).

### 2.6. Map-Based Cloning

A map-based cloning approach was used to identify the *dfin* locus. The *dfin* mutants in the Col-0 background isolated from the activation tagging mutant population were crossed to the L*er* wild-type. Nineteen F1 hybrid plants were grown on a growth medium for further analysis. For root growth assays, seven-day-old F1 seedlings were transferred to a solid medium containing 10 μM DFPM and monitored to observe root growth arrest after six more days. A population of Col-0 *dfin* × L*er* F2 seeds was collected from the self-pollination of the F1 hybrids, and their root growth phenotypes were determined. For the initial mapping, 48 homozygous wild types were isolated from the F2 population and genotyped with 22 mapping markers, spaced roughly every 20 cM apart on the five chromosomes. An additional 31 F2 plants and mapping markers were used to narrow down the candidate to the 50 Kb region. The coding region of *VICTR* was sequenced by using multiple primer pairs (Table S1), and the single-nucleotide mutations in multiple *dfin* mutants from independent seed pools were identified to confirm whether the corresponding *dfin* mutations were in the *VICTR* locus.

## 3. Results

### 3.1. DFPM Derivatives Have Specific Effects on Root Growth

**Figure 1:**
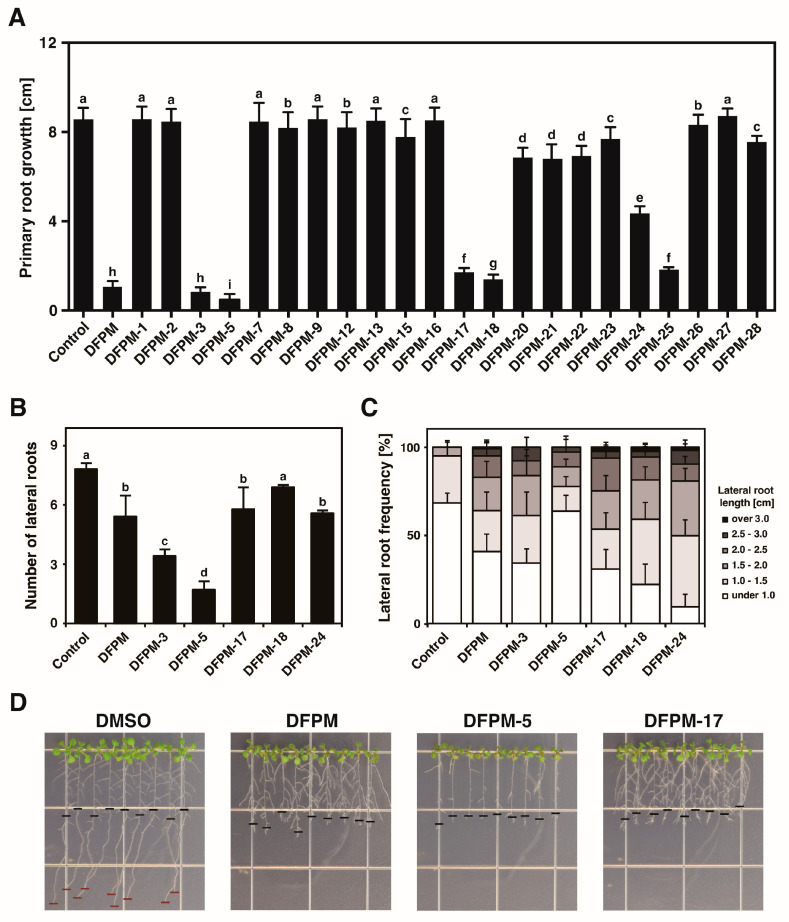
Effects on the primary and lateral root growth by the DFPM derivatives. (**A**) The DFPM-induced primary root growth arrest phenotype was compared to roots treated with twenty-two DFPM derivatives. Seven-day-old Col-0 seedlings were treated with 10 μM of DFPM or DFPM derivatives and monitored for their growth after six days. Several DFPM derivatives produced significant primary root growth arrest phenotypes in the Col-0 wildtype. (**B**) The lateral root number and (**C**) lateral root frequency of plants treated with selected compounds were examined. (**D**) Representative photographs of Col-0 seedlings grown on MS media containing DMSO, DFPM, DFPM-5, and DFPM-17. The black horizontal bars on the root tips mark the starting point when the plants were transferred to the plates with designated chemicals. Treatment with 10 μM DMSO was used as a control. Data represent mean values ± SD (*n* = 3, each with 12 seedlings). Different letters on top of the error bars indicate significant differences as determined by one-way ANOVA with Tukey’s post-test (*p* < 0.001).

The small compound DFPM inhibited the ABA signal transduction and caused primary root growth arrest in an accession-specific way by targeting the TNR protein *VICTR* [13,29]. The root growth arrest phenotype was assessed for the twenty-two chemical compounds structurally similar to DFPM to define critical structural motifs required for triggering the DFPM-induced root growth arrest and isolate novel derivative compounds containing more specific functions than DFPM (Figure S1). Seven-day-old seedlings grown from a normal growth medium were transferred to the plates with 10 μM of each of the chemicals and monitored for the production of any root growth arrest phenotype for a week. Overall, many derivatives lost their activity on the primary root growth arrest, even those with a slight structural modification of the original DFPM. On the other hand, several derivatives produced significant root growth arrests similar to the phenotypes produced by DFPM (Figure 1A). Based on the primary root growth phenotype, six notable compounds showing similar biological activity to the level of original DFPM were identified: DFPM-3, -5, -17, -18, -24, and -25. While DFPM-25 had poor synthesis efficiency, the other five small compounds were further analyzed. The lateral root phenotypes, including the total numbers and lengths of lateral roots for the five candidate compounds, were analyzed further. Treatments with the selected DFPM derivatives reduced the total number of lateral roots compared to the DMSO control (Figure 1B). The DFPM-17, -18, and -24 treatments decreased the number of lateral roots while increasing the average later root length compared to the DMSO control. Application of DFPM-3 and -5 exhibited stronger inhibitory effects on the lateral root formation compared to the DFPM. Among the compounds analyzed, DFPM-5, a precursor of the original DPFM, produced the highest inhibitory effect on the primary root growth (Figure 1A) and the number and length of lateral roots (Figure 1B,C).

### 3.2. Selected Chemicals Affect Expression Patterns of the ABA-Responsive Genes and the Pathogen-Responsive Genes

**Figure 2:**
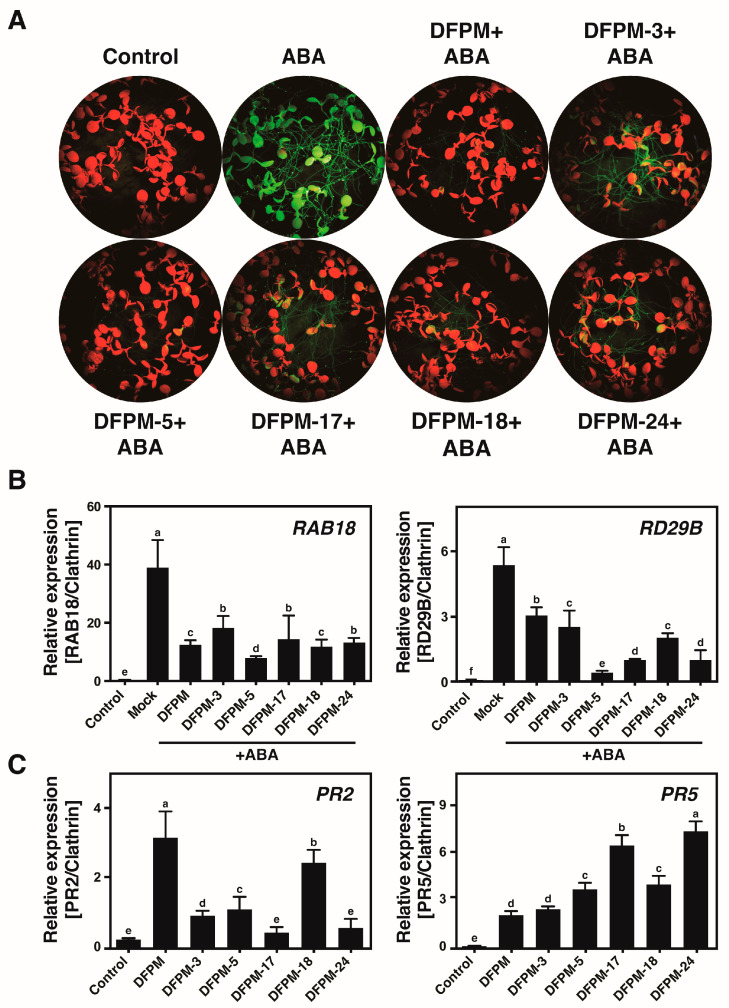
Selected DFPM derivatives inhibit the ABA induction of gene expression and stimulate the induction of pathogen-responsive gene expression. (**A**) Treatments with the DFPM derivatives reduced the ABA induction of GFP expression in *RAB18-GFP* reporter lines. Two-week-old transgenic seedlings were pre-treated with 40 µM of DFPM derivatives or DMSO for 6 h and then incubated in 10 µM ABA for 10 h. The same concentration of DMSO without ABA was used as a control. (**B**) DFPM derivatives in the inhibition of the ABA-induced gene expression were measured by quantitative PCR. Seedlings were pre-treated with 30 μM DFPM derivatives or DMSO (mock treatment) for an hour and then incubated with 10 µM ABA for five hours. DMSO treatment without ABA was used as a control. (**C**) DFPM derivatives in the induction of the pathogen-responsive gene expression were measured by quantitative PCR. Seedlings were treated with 30 μM DFPM derivatives or DMSO (control) for six hours. Data represent mean values ± SD (*n* = 3). Different letters on top of the error bars indicate significant differences as determined by one-way ANOVA with Tukey’s post-test (*p* < 0.001).

DFPM disrupts the ABA-induced gene expression through interference in ABA signaling [13]. The ABA induction of *RAB18* was examined together with candidate chemicals using the *RAB18* promoter–GFP reporter lines to determine if the selected DFPM derivatives could inhibit the induction of the ABA-responsive genes. Treatments with the five DFPM derivatives reduced the ABA-induced *RAB18* expression in the *GFP* reporter lines (Figure 2A). Downregulation of the ABA-responsive genes, including *RAB18* and *RD29B*, was also confirmed by quantitative RT-PCR analyses in Col-0 (Figure 2B). In particular, DFPM-5 showed the strongest inhibitory effect on ABA-responsive gene expression, which was also observed in root growth analysis (Figure 1A,B and Figure 2B). DFPM also induces plant immunity-related gene expression by activating TNL-derived immune signaling [29]. The selected DFPM derivatives produced inductions of the pathogen-responsive *PR2* and *PR5* genes (Figure 2C). The increase in *PR2* gene expression by most small compounds was smaller than that by DFPM, whereas *PR5* gene expression by DFPM-5, -17, -18, or -24 was upregulated significantly compared to that by DFPM (Figure 2C).

### 3.3. Selected DFPM Derivatives May Interfere with Root Growth through the Same Signal Transduction Pathway Controlled by DFPM

**Figure 3:**
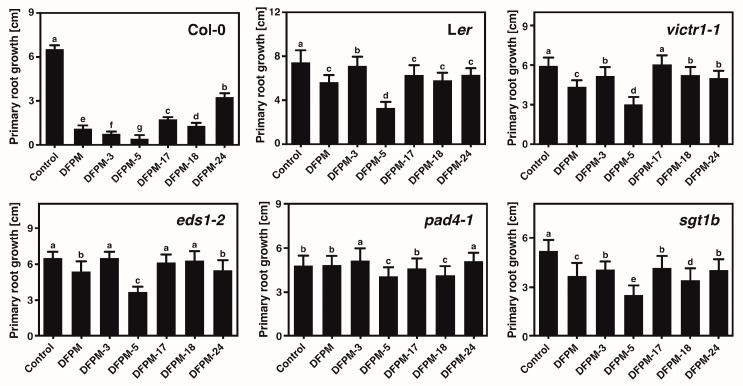
DFPM-derivative-induced signal transduction requires early components of the TNL-triggered immune signaling. L*er* and *victr1-1* defective in the function of *VICTR* and pathogen mutants, such as *eds1-2*, *pad4-1*, and *sgt1b*, produced no significant growth arrest in response to the DFPM derivatives. All mutants were tested in the Col-0 background. Data represent mean values ± SD (*n* = 3, each with 12 seedlings). Different letters on top of the error bars indicate significant differences as determined by one-way ANOVA with Tukey’s post-test (*p* < 0.001).

TNL-triggered immune signaling components, including *EDS1*, *PAD4*, *SGT1b*, *RAR1*, and *VICTR*, are required for DFPM root growth arrest and the inhibition of ABA signal transduction. This study examined whether the selected DFPM derivatives cause a DFPM-induced root growth response in an EDS1/PAD4-dependent signaling pathway. The inhibitory effect on primary root growth was examined in several defense-signaling mutants, including *eds1-2*, *pad4-1*, *sgt1b*, and *victr1-1*. Although the Col-0 accession containing a functional *VICTR* allele was sensitive to the five DFPM derivatives, Ler and *victr1-1*, with defects in the function of *VICTR*, exhibited increased primary root length in response to the DFPM derivatives when compared to Col-0 (Figure 3). In addition to the need for the functional *VICTR* in inhibiting root growth, the inhibition of root growth by the selected DFPM derivatives was impaired in *eds1-2*, *pad4-1*, and *sgt1b* (Figure 3). These results suggest that the selected DFPM derivatives might activate the same signaling pathway required for DFPM to generate the compound-triggered root growth arrest.

### 3.4. Selected DFPM Derivatives Cause Accession-Specific Root Growth Arrest in Col-0, Nie1.2, and Leo1

**Figure 4:**
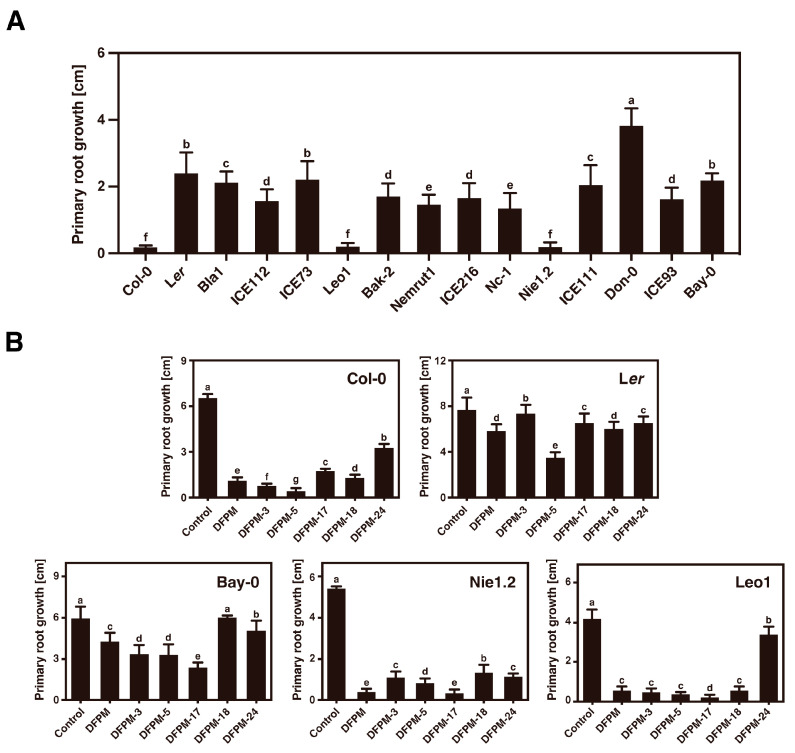
Differential effects of the DFPM derivatives on primary root growth in Arabidopsis accessions. (**A**) The DFPM-induced root growth arrest phenotype was examined for eleven other Arabidopsis accessions. Seven-day-old Arabidopsis seedlings were treated with 10 μM of DFPM, and their growth was monitored after four days. DFPM caused a root growth arrest in the Col-0 background and in the Nie1.2 and Leo1 backgrounds in an accession-specific manner. (**B**) Nie1.2 and Leo1 plants were sensitive to the selected DFPM derivatives, producing significantly shorter primary roots than the other accessions. Data represent mean values ± SD (*n* = 3, each with 12 seedlings). Different letters on top of the error bars indicate significant differences as determined by one-way ANOVA with Tukey’s post-test (*p* < 0.001).

DFPM generates an accession-specific root growth arrest in Col-0. This phenotypic response relies on natural variation in the TNL receptor *VICTR*. A previous study showed that only Col-0 accession displayed complete sensitivity in the DFPM-induced root growth arrest among 50 other Arabidopsis accessions [29]. For this study, the DFPM-induced root growth assay was performed for 14 more Arabidopsis accessions that specifically have sequence similarities to the genome of the Col-0 accession (Figure S2). As a result, two other Arabidopsis accessions, Nie1.2 and Leo1, that produced significant root growth inhibition in response to DFPM were isolated (Figure 4A). The Nie1.2 and Leo1 plants treated with the selected DFPM derivatives exhibited impaired primary root growth similar to that seen with DFPM treatment, suggesting potential natural genetic variations that cause DFPM-derivative-induced root growth arrest in Nie1.2 and Leo1 as in Col-0 (Figure 4B).

### 3.5. Twelve Natural Variation Sites in the TNL Receptor VICTR Are Required for Triggering the DFPM-Induced Root Growth Arrest

**Figure 5:**
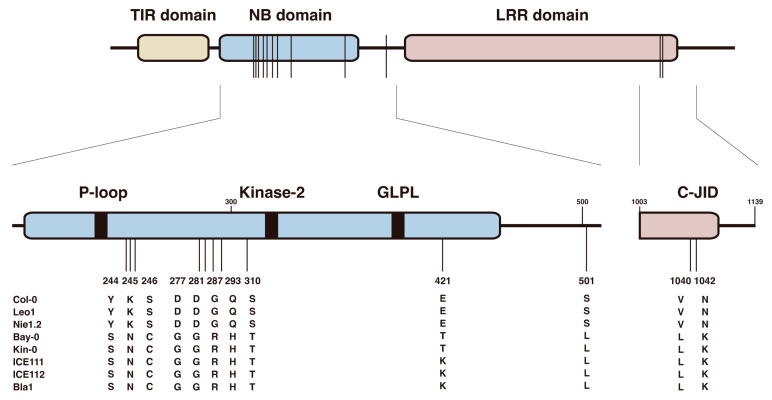
Specific amino acid polymorphisms of *VICTR* are responsible for the DFPM-induced root growth response. From the sequence analysis, 12 amino acid polymorphisms of *VICTR*, which are unique to only DFPM-sensitive accessions, were identified. The specific residues of *VICTR* could be a critical motif for generating the DFPM-induced root growth arrest response. Vertical lines indicate sequence positions where the DFPM-sensitive accessions differ from DFPM-resistant accessions.

A previous study reported that amino acid sequence comparison deduced from the *VICTR* cDNA sequences of Col-0, Bay-0, and Kin-0 identified multiple Col-0-specific amino acid polymorphisms within the coding region of *VICTR^Col^* [29]. The full-length *VICTR* genomic DNA sequences from the eight Arabidopsis accessions were compared to define which amino acids of *VICTR^Col^* are critically involved in triggering the compound-induced root growth arrest. *VICTR* genomic DNA fragments were amplified from eight Arabidopsis accessions: Col-0, Leo1, Nie1.2, Bay-0, Kin-0, Bla1, ICE111, and ICE 112. The deduced amino acid sequences of the amplified DNA fragments from each Arabidopsis accession were aligned using the Clustal Omega tool (Figure S2). Sequence analysis showed that 12 natural variations found in the *VICTR^Col^* were also identified in other DFPM-sensitive Arabidopsis accessions, Leo1 and Nie1.2 (Figure 4 and Figure S2). Among these variants, most polymorphisms were located in the NB domain. Other sequence changes were found in the LRR domain and linker region between the NB and LRR domains (Figure 5).

During our forward genetic screening, *dfin* (*DFPM-insensitive to root growth*) mutants were isolated from the activation-tagged mutant population. From the initial screening of 6810 activation-tagged mutant plants, 19 *dfin* mutants that showed defects in the DFPM-mediated root growth arrest were isolated (Figure S3A). A map-based cloning approach was used to identify the *dfin* locus, and 12 *dfin* mutants were found near the *VICTR* locus (Figure S3B). The corresponding *dfin* mutations were in the *VICTR* locus which was confirmed by sequencing the coding region of *VICTR*. The *dfin* mutant contains either G-to-C or T-to-A point mutations, leading to V1040L or N1042K substitutions in the LRR domain, respectively, which are specific polymorphisms essential for the DFPM-mediated root growth arrest (Figure S3C). Therefore, a critical residue of the *VICTR* gene that is a non-conserved site in the *R* gene could be a critical motif for generating the DFPM-induced root growth response.

## 4. Discussion

Many thioamide compounds are beneficial, especially with biological activities, such as antimicrobial, anticancer, antituberculosis, and anthelmintic properties [32,33,34,35,36]. DFPM, which belongs to the thioamide class, exhibits diverse activities, including inhibiting ABA signaling and primary root meristem activity by activating plant immune signaling. Twenty-two chemical compounds structurally similar to DFPM were synthesized by modifying the phenyl and amide groups of DFPM. Five notable compounds with enhanced inhibitory activity on the primary root growth were initially selected: DFPM-3, -5, -17, -18, and -24. These compounds can be classified into two groups depending on their structural changes resulting from a single-atom substitution. DFPM-17, -18, -24, and original DFPM belong to thioamides in which a sulfur atom replaced the carbonyl oxygen. DFPM-3 and DFPM-5, containing the carbonyl oxygen, may function as precursors of the DFPM-17 and original DFPM, respectively. Root growth inhibition assays showed that the precursors, DFPM-3 and DFPM-5, had enhanced inhibitory effects on both primary and lateral root growth compared to sulfur-containing thioamide compounds, DFPM-17 and DFPM (Figure 1). In particular, DFPM-5, as a precursor of the original DFPM, exhibited the strongest inhibitory effect on root growth among all DFPM derivatives. The structural difference between amide and thioamide allows changes in many physicochemical properties, including hydrogen-bonding propensity, conformational flexibility, and spectroscopic and electrochemical properties that contribute to protein folding and stability [32,37]. These results suggest that subtle changes to amide interactions from the single-atom substitution may allow an enhanced inhibitory effect on root growth.

DFPM negatively affects the ABA signaling pathway by activating TNL-mediated immune signaling [29]. Similar to DFPM, the selected DFPM derivatives inhibited the ABA induction of *RAB18* expression, while the ABA induction of *RB29B* expression was more strongly inhibited by the selected DFPM derivatives than by DFPM (Figure 2). Furthermore, the selected compounds enhanced the pathogen-responsive gene expression, suggesting that the selected DFPM derivatives negatively regulate ABA signal transduction by activating the plant immune responses, as in the case of DFPM. Among the DFPM derivatives, DFPM-5 produced the strongest inhibitory effect on both ABA signaling genes, which is consistent with the result of root growth assays. In addition, DFPM-5 thioamides appeared to be more effective in inhibiting the ABA-responsive genes and the activation of pathogen-responsive genes.

The DFPM response producing a root growth arrest requires the TNL-mediated immune signaling components, including *EDS1*, *PAD4*, *SGT1b*, *RAR1*, and *VICTR*. The selected DFPM derivatives did not inhibit the root growth of L*er*, *victr1-1*, and defense signaling mutants, indicating that the selected DFPM derivatives require a functional *VICTR* allele along with *EDS1*, *PAD4*, and *SGT1B* to trigger compound-induced root growth arrests. Overall, the selected DFPM derivatives generate compound-induced root growth inhibition, possibly by activating the same signaling pathway that DFPM activates. *EDS1* acts as a central regulatory node in TNL-mediated plant immunity with its sequence-related signaling partners, *PAD4* and *SAG101*, which can function in separate pathways to generate basal resistance and ETI [20,21,22]. Among the DFPM derivatives, DFPM-18 enhanced *PR2* gene expression the most compared to other DFPM derivatives (Figure 2C). Furthermore, DFPM-18 showed an enhanced root growth inhibitory effect in *pad4-1* when compared to the original DFPM (Figure 3). Hence, the DFPM-18 response can cause root growth arrest through a plant immune signaling pathway partially independent of *PAD4*. In particular, DFPM-5 inhibited root growth and ABA-induced gene expression the most strongly. This inhibition was also observed, even in the background of mutations essential for DFPM signaling, suggesting that DFPM5-induced response may be generated by an independent pathway different from the signal transduction pathway by DFPM. DFPM may trigger the TNL-mediated signal transduction by acting as a pathogen-associated molecule [29]. This hypothesis proposes that the only structural difference between DFPM-5 and the original DFPM, the carbonyl oxygen, can be a critical site for improving the functional interaction between compounds and a TNL receptor.

The plant NB-LRR genes have evolved to recognize pathogen effectors, leading to the activation of plant defense response against various classes of pathogens. In many cases, the central NB domain functions as a molecular switch activating downstream signal transduction, while the LRR is responsible for effector recognition specificity and auto-inhibition of NLRs [38,39,40]. Comparative sequence analyses of the *VICTR* region between the eight Arabidopsis accessions revealed 12 natural variation sites in the TNL receptor *VICTR* required for triggering DFPM-induced root growth arrest (Figure 5). Among these variants, V1040L and N1042K substitutions within the LRR domain are consistently found in the isolated new *dfin* mutants (Figure S3). These two polymorphisms are located at the C-terminal end of the LRR domain, termed the C-terminal jelly roll/Ig-like domain (C-JID), which is essential for effector recognition and TNL-mediated innate immunity [41,42]. The deletion or mutation in the C-JID region of TNL genes, such as the Arabidopsis *RPS4* gene and the tobacco *ROQ1* and *N* genes, failed to trigger a hypersensitive response [42,43]. The C-JID core consists of approximately 130 amino acids, which form two β-sheets and fold into a β-sandwich structure [41,42,43]. This β-sandwich core and its loop regions are responsible for pathogen effector recognition. For example, a loop region between β-strands 7 and 8 of the RPP1C-JID interacts with the pathogen effector ATR1 through hydrogen bonds and hydrophobic interactions [43]. The two polymorphisms V1040 and N1042 identified in victr mutant alleles are predicted to locate in a loop region connecting β-strands within the C-JID of *VICTR*. Therefore, these single-nucleotide polymorphisms may also have a potential role in the specific recognition of effectors in the DFPM-mediated signal transduction, such as binding a ligand or other protein. Further research on the structures of the full-length VICTR protein and multiple VICTR variants is necessary to understand the interaction between the TNL protein VICTR and target effectors. Such structural information provides insight into specific mechanisms of direct effector recognition via the C-JID and LRR domains in the DFPM-mediated immune response.

## 5. Conclusions

This study reported that novel chemical compounds structurally similar to DFPM effectively inhibit ABA signaling transduction by activating the effector-triggered immune signaling pathway. In addition, the selected DFPM compounds induced a rapid root growth arrest via the TNL receptor *VICTR* in an *EDS1/PAD4/RAR1/SGT1B*-dependent or -independent manner. Finally, multiple unique amino acid polymorphisms were identified in the VICTR locus required for the root growth arrest phenotype caused by DFPM or the selected DFPM derivatives. The existence of a stronger version of DFPM due to chemical modification suggests that specific functional residues may determine the nature of DFPM activities. The isolation of novel compounds, such as DFPM-5, including a chemical genetic screening for genes involved in the corresponding signaling pathway will provide a stronger tool for unraveling the mechanisms of DFPM that induce the interaction of abiotic and biotic stress signal transductions. 

## Data Availability

All the obtained data from the research are presented in the manuscript and supplementary material.

## Supplementary Materials

The following supporting information can be downloaded at: https://www.mdpi.com/article/10.3390/life13091797/s1. Figure S1: Chemical structures of DFPM derivatives; Figure S2: Sequence comparison of *VICTR* alleles from 8 different Arabidopsis accessions; Figure S3: Isolation of new *victr* mutant alleles that reveal key residues for the DFPM-induced root arrest; Table S1: The primers used in this experiment.
