# Supplementary material for: Novel Compounds Derived from DFPM Induce Root Growth Arrest through the Specific VICTR Alleles of Arabidopsis Accessions
Source: Life (Basel). 2023 Aug 23;13(9):1797. doi: 10.3390/life13091797 (PMC10532556; doi:10.3390/life13091797)
Supplement: Supplementary file 1 [file life-13-01797-s001.zip › Figure S1.pdf]

**DFPM-1** 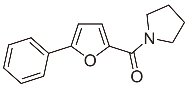 [5-Phenyl-2-furyl](1-pyrrolidinyl)methanone	**DFPM-15** 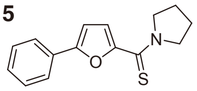 [5-Phenyl-2-furyl](1-pyrrolidinyl)methanethione
**DFPM-2** 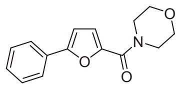 [5-Phenyl-2-furyl](1-morphoridinyl)methanone	**DFPM-16** 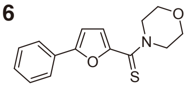 [5-Phenyl-2-furyl](1-morphoridinyl)methanethione
**DFPM-3** 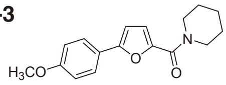 [5-(4-methoxy)-2-furyl](1-piperidinyl)methanone	**DFPM-17** 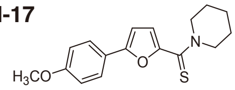 [5-(4-methoxy)-2-furyl](1-piperidinyl)methanethione
**DFPM-4** 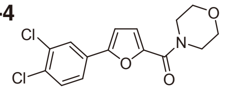 [5-(3,4-Dichlorophenyl)-2-furyl](1-morphoridinyl)methanone	**DFPM-18** 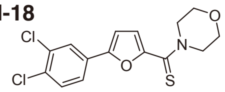 [5-(3,4-Dichlorophenyl)-2-furyl](1-morphoridinyl)methanethione
**DFPM-5** 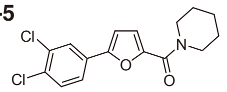 [5-(3,4-Dichlorophenyl)-2-furyl](1-piperidinyl)methanone	**DFPM-19 (DFPM)** 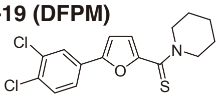 [5-(3,4-Dichlorophenyl)-2-furyl](1-piperidinyl)methanethione
**DFPM-6** 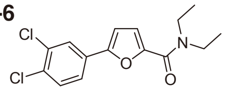 N,N-Dimethyl-[5-(3,4-Dichlorophenyl)-2-furyl]amide	**DFPM-20** 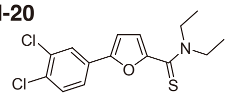 N,N-Dimethyl-[5-(3,4-Dichlorophenyl)-2-furyl]thioamide
**DFPM-7** 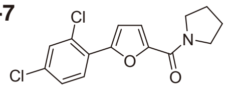 [5-(2,4-Dichlorophenyl)-2-furyl](1-pyrrolidinyl)methanone	**DFPM-21** 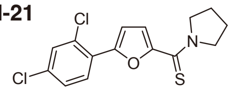 [5-(2,4-Dichlorophenyl)-2-furyl](1-pyrrolidinyl)methanethione
**DFPM-8** 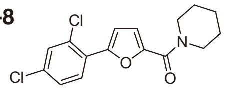 [5-(2,4-Dichlorophenyl)-2-furyl](1-piperidinyl)methanone	**DFPM-22** 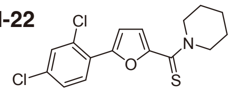 [5-(2,4-Dichlorophenyl)-2-furyl](1-piperidinyl)methanethione
**DFPM-9** 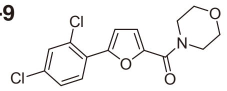 [5-(2,4-Dichlorophenyl)-2-furyl](1-morphoridinyl)methanone	**DFPM-23** 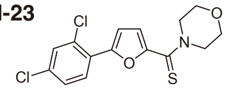 [5-(2,4-Dichlorophenyl)-2-furyl](1-morphoridinyl)methanethione
**DFPM-10** 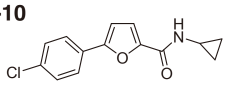 N-cyclopropyl-[5-(4-chlorophenyl)-2-furyl]amide	**DFPM-24** 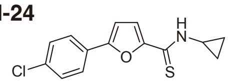 N-cyclopropyl-[5-(4-chlorophenyl)-2-furyl]thioamide
**DFPM-11** 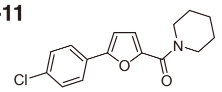 [5-(4-chlorophenyl)-2-furyl](1-piperidinyl)methanone	**DFPM-25** 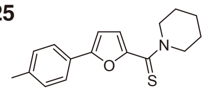 [5-(4-chlorophenyl)-2-furyl](1-piperidinyl)methanethione
**DFPM-12** 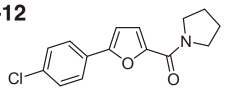 [5-(4-chlorophenyl)-2-furyl](1-pyrrolidinyl)methanone	**DFPM-26** 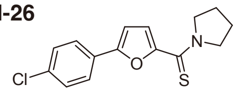 [5-(4-chlorophenyl)-2-furyl](1-pyrrolidinyl)methanethione
**DFPM-13** 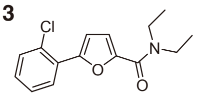 N,N-Dimethyl [5-(2-chlorophenyl)-2-furyl]amide	**DFPM-27** 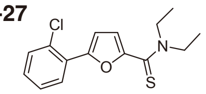 N,N-Dimethyl [5-(2-chlorophenyl)-2-furyl]thioamide
**DFPM-14** 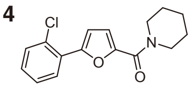 [5-(2-chlorophenyl)-2-furyl](1-piperidinyl)methanone	**DFPM-28** 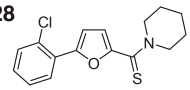 [5-(2-chlorophenyl)-2-furyl](1-piperidinyl)methanethione

Figure S1. Chemical structures of DFPM derivatives.

Twenty-two chemical compounds structurally similar to DFPM were synthesized by modifying the phenyl and amide groups. Each of the 14 compounds contained within a left column is a precursor of the compound on the right side. The original DFPM is designated here as DFPM-19. The DFPM-4, -6, -10, -11, and -14 were excluded from further analysis because of their low yield in synthesis.
