# Supplementary material for: Novel Compounds Derived from DFPM Induce Root Growth Arrest through the Specific VICTR Alleles of Arabidopsis Accessions
Source: Life (Basel). 2023 Aug 23;13(9):1797. doi: 10.3390/life13091797 (PMC10532556; doi:10.3390/life13091797)
Supplement: Supplementary file 1 [file life-13-01797-s001.zip › Figure S3.pdf]

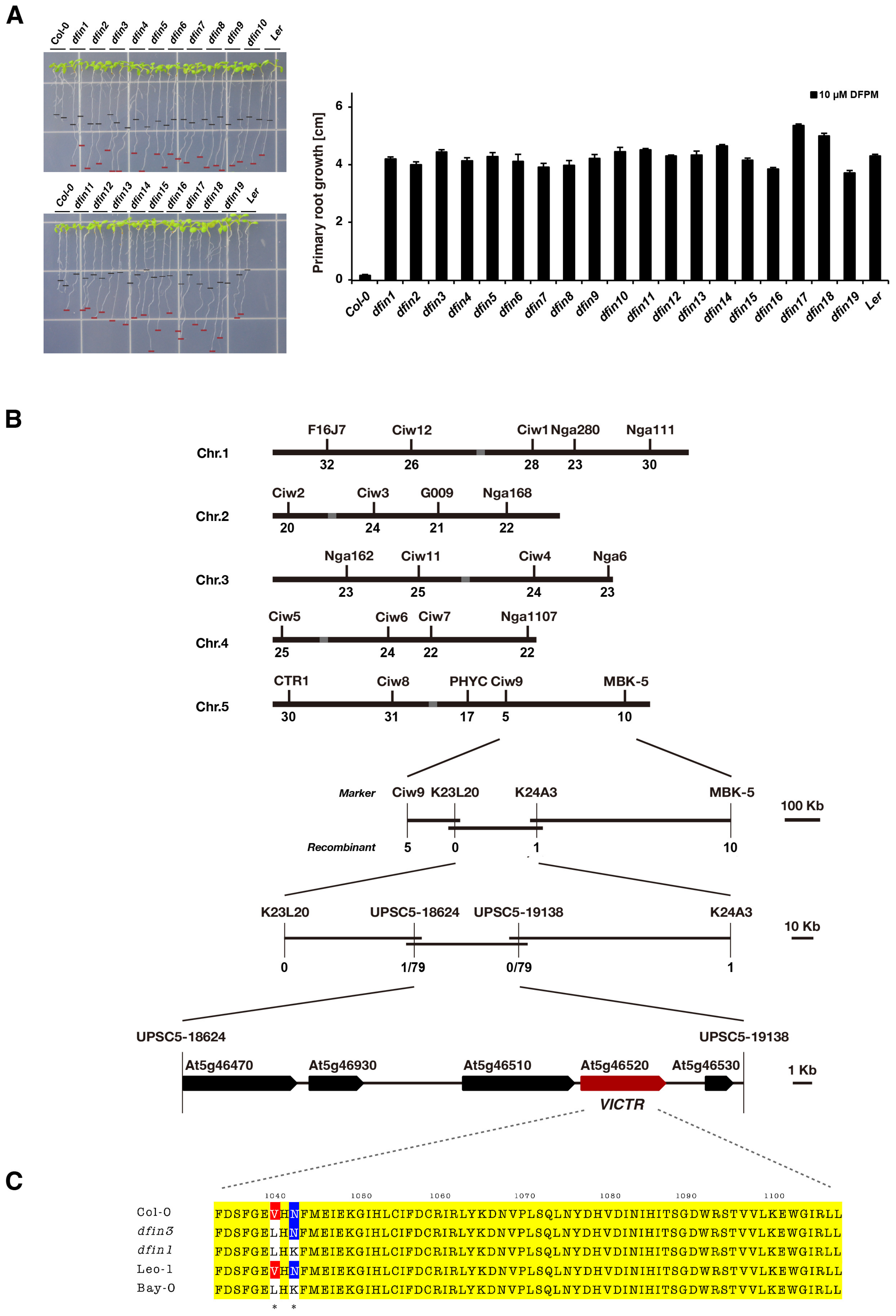

Group	Description	Mutants
I	V1040L	*dfin3, dfin8, dfin10, dfin11, dfin13, dfin14*
II	V1040L, N1042K	*dfin1, dfin2, dfin4, dfin5, dfin7, dfin9*

Figure S3. Isolation of new *victr* mutant alleles that reveal key residues for the DFPM-induced root arrest.

(**A**) Nineteen *dfin* (DFPM-insensitive to root growth) mutants were isolated from the activation tagging mutant population. Seven-day-old Col-0 seedlings grown in a normal plate medium were transferred to a new plate containing 10 μM DFPM. Each primary root of the seedlings was monitored to observe any root growth defect after six days. The data represent the mean values ± SD (*n*=3, each with 12 seedlings). (**B**) Several *dfin* mutations were positioned in the TNL gene *VICTR* by a map-based cloning approach. Initial mapping showed that the *dfin* locus displayed a linkage to the ciw9 marker on chromosome V. The additional markers between ciw9 and MBK-5 were used to narrow down the candidate to the 50 Kb region. The numbers under the linkage map indicate the number of recombinants. (**C**) Entire *VICTR* genes of 19 *dfin* mutants were sequenced and compared with that of the wild-type Col-0 and other Arabidopsis accession Leo-1 and Bay-0 plants. Among the dfin mutants analyzed, 12 *dfin* mutants contain mutations in the *VICTR* gene that were recognized as new *victr* mutant alleles. New *vitcr* mutant alleles were classified into two different groups based on their nucleotide changes. Six *dfin* mutants of the first group carry a single point mutation, valine(1040) to leucine in the VICTR C-terminus region. The other six *dfin* mutants belonging to the second group contained two nucleotide substitutions, asparagine(1042) to lysine and the group 1 mutation, Val1040Leu.
