# Supplementary material for: Novel Compounds Derived from DFPM Induce Root Growth Arrest through the Specific VICTR Alleles of Arabidopsis Accessions
Source: Life (Basel). 2023 Aug 23;13(9):1797. doi: 10.3390/life13091797 (PMC10532556; doi:10.3390/life13091797)
Supplement: Supplementary file 1 [file Figure S2.pdf › Figure S2.pdf]

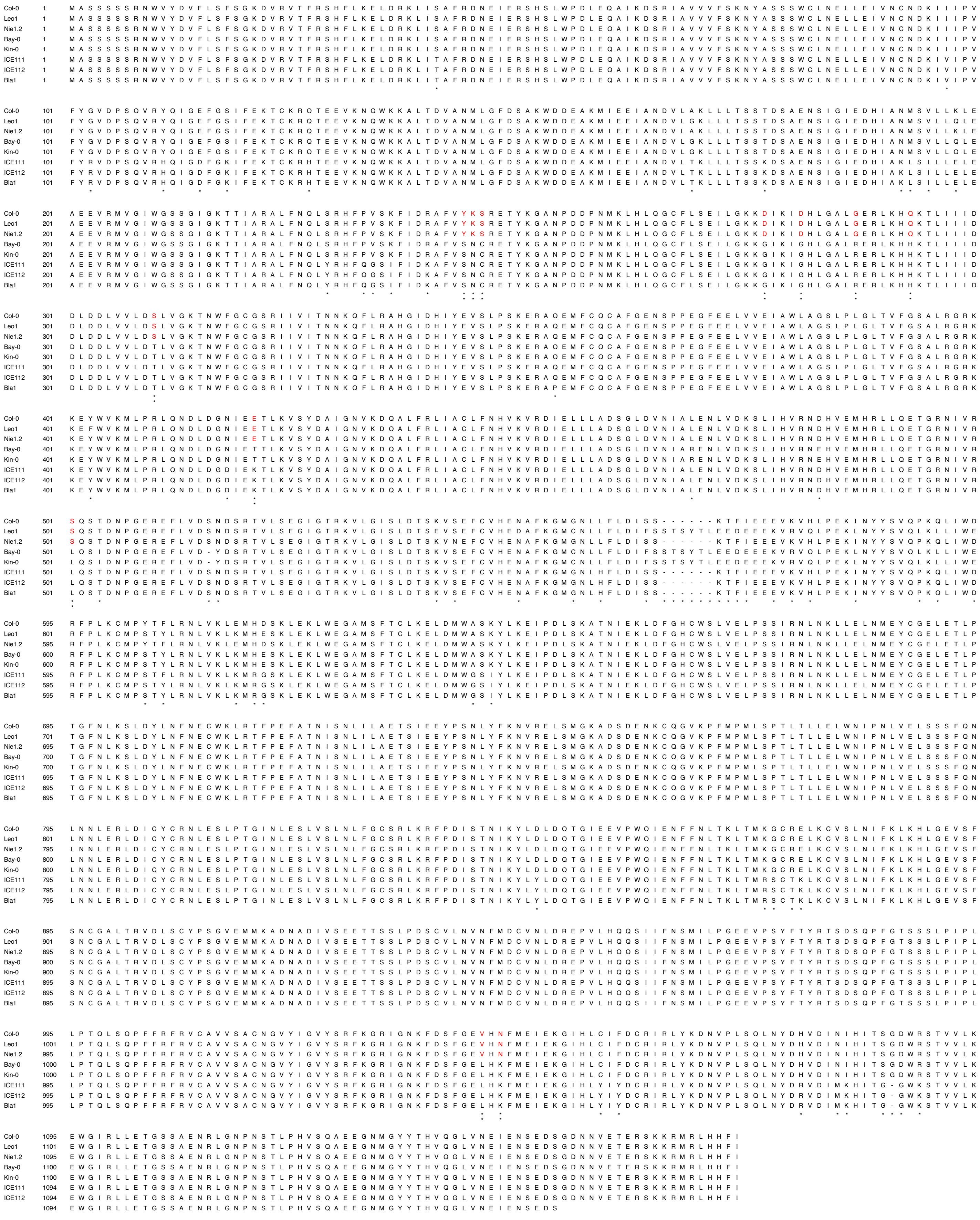

Figure S2. Sequence comparison of *VICTR* alleles from 8 different Arabidopsis accessions.
Each amino acid sequence was deduced by sequencing of full-length *VICTR* genomic DNAs from eight different Arabidopsis accessions. The sequence alignment was generated using Clustal Omega. Amino acid polymorphisms of *VICTR* specifically found in DFPM-sensitive accessions are marked by asterisks.
